# Spectroscopic Analysis of the Extracellular Matrix in Naked Mole-Rat Temporomandibular Joints

**DOI:** 10.3390/gels11060414

**Published:** 2025-05-30

**Authors:** Tetsuya Adachi, Hayata Imamura, Toyonari Yaji, Kentaro Mochizuki, Wenliang Zhu, Satoru Shindo, Shunichi Shibata, Keiji Adachi, Toshiro Yamamoto, Fumishige Oseko, Osam Mazda, Kyoko Miura, Toshihisa Kawai, Giuseppe Pezzotti

**Affiliations:** 1 Department of Dental Medicine, Graduate School of Medical Science, Kyoto Prefectural University of Medicine, Kamigyo-ku, Kyoto 602-8566, Japan; d2871002@edu.kit.ac.jp (H.I.); keiji922@koto.kpu-m.ac.jp (K.A.); yamamoto@koto.kpu-m.ac.jp (T.Y.); foseko@koto.kpu-m.ac.jp (F.O.); 2 Department of Immunology, Graduate School of Medical Science, Kyoto Prefectural University of Medicine, Kamigyo-ku, 465 Kajii-cho, Kyoto 602-8566, Japanpezzotti@hirakata.kmu.ac.jp (G.P.); 3 Ceramic Physics Laboratory, Kyoto Institute of Technology, Sakyo-ku, Matsugasaki, Kyoto 606-8585, Japan; wlzhu@kit.ac.jp; 4 Synchrotron Radiation Center, Ritsumeikan University, 1-1-1 Noji-Higashi, Kusatsu, Shiga 525-8577, Japan; tyv27078@fc.ritsumei.ac.jp; 5 Department of Pathology and Cell Regulation, Graduate School of Medical Science, Kyoto Prefectural University of Medicine, Kamigyo-ku, Kyoto 602-8566, Japan; k-mochi@koto.kpu-m.ac.jp; 6 College of Dental Medicine, Nova Southeastern University, 3200 South University Drive, Fort Lauderdale, FL 33328, USA; sshindo1@nova.edu (S.S.); tkawai@nova.edu (T.K.); 7 Department of Anatomy, School of Dentistry, Health Sciences University of Hokkaido, 1757 Kanazawa, Tobetsu-cho, Ishikari-gun, Hokkaido 061-0293, Japan; sshibataanat@hoku-iryo-u.ac.jp; 8 Department of Aging and Longevity Research, Faculty of Life Sciences, Kumamoto University, Kumamoto 860-8556, Japan; miurak@kumamoto-u.ac.jp; 9 Biomedical Engineering Center, Kansai Medical University, 1-9-11 Shin-machi, Hirakata, Osaka 573-1191, Japan

**Keywords:** extracellular matrix, glycosaminoglycan, hyaluronic acid, naked mole-rat, anti-aging

## Abstract

Naked mole-rats are extremely long-living rodents with a maximum lifespan of 37 years, and their cellular aging and tissue aging are almost nonexistent. Therefore, in this study, we aim to analyze the extracellular matrix of the temporomandibular joint (TMJ) of naked mole-rats at the molecular level and explore the molecules involved in anti-aging and their localization. Micro-computed tomography (CT) scans revealed increased mineral density and wear of the mandibular condyle in aged mice. Conversely, CT scans did not reveal wear of the mandibular condyle in naked mole-rats, and histological analysis did not reveal wear of the articular disk. Using various spectroscopies and artificial intelligence (AI), we found that the articular disk of naked mole-rats is composed of a cartilage-like layer with hyaluronic acid and collagen fibers with varying orientations, which is thought to have relieved mechanical stress and have protected the mandibular condyle. These results suggest that not only the amount, but also the spatial distribution of the extracellular matrix is important for the anti-aging properties of the TMJ, and may contribute to elucidating the pathology of TMJ disorders and other degenerative conditions and developing therapeutic drugs.

## 1. Introduction

Although the average life expectancy of Japanese people has reached record highs year after year, the difference in healthy life expectancy (the period during which one can lead an independent and healthy life) is more than 10 years. Therefore, the extension of a healthy life expectancy is an urgent issue in Japan, which is a super-aging society. The presence or absence of the deterioration of oral function in the elderly is listed as one of the key items for care prevention. Oral organs involved in mastication include the jaw bone, teeth, temporomandibular joints (TMJ), masticatory muscles, etc. TMJ dysfunction causes pain and limits the movement of the jaw joint [1,2].

According to the NIH, the global prevalence of symptomatic TMJ is typically estimated at 5–12%. [https://www.nidcr.nih.gov/research/data-statistics/facial-pain/prevalence, accessed on 28 April 2025]. Not all TMJ disorders require medical intervention, but the associated medical costs and loss of economic productivity are estimated to be USD 4 billion per year [3]. A decline in masticatory function (including TMJ dysfunction and missing teeth) causes eating and swallowing disorders, malnutrition, and general muscle mass and muscle weakness (sarcopenia).

Decreased masticatory function due to aging decreases the muscle activity of the masticatory muscles and degeneration of the TMJ, therefore preventing degenerative changes, as it will lead to the prevention of sarcopenia and the extension of a healthy life expectancy.

**Figure 1:**
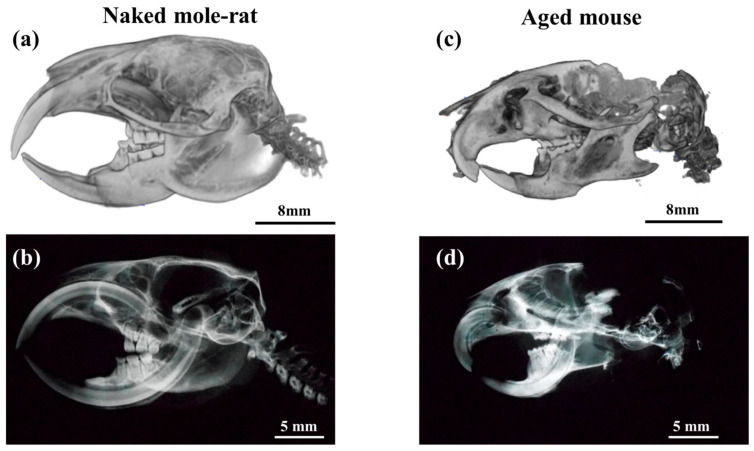
(**a**,**c**) Representative three-dimensional reconstructions and (**b**,**d**) plain radiographs of the skulls of a naked mole-rat and aged mouse.

Vapniarsky et al. subcultured costal chondrocytes to produce cultured articular disks with mechanical properties equivalent to those of scaffold-free natural articular disks and applied these to the treatment of temporomandibular joint disorders [3]. However, because this method involves the transplantation of autologous cultured cartilage, a large defect is created at the harvest site. Therefore, there is still no fundamental treatment to repair the cartilage of the TMJ and articular disks. We therefore focused on naked mole-rats, which are ultra-long-living rodents. Naked mole-rats show marked resistance to aging, with a maximum lifespan of 37 years, with almost no signs of aging or deterioration in their various tissues and organs. Naked mole-rats have a distinctive appearance, such as long incisors and a lack of body hair (Figure 1a,b). Naked mole-rats are unique in that not only their tooth crowns, but also their tooth roots are longer than those of mice (Figure 1c,d).

Furthermore, it is thought that naked mole-rats, like humans, utilize a large proportion of the cerebral cortex during oral sensations. Therefore, elucidation of this unique physiological function would lead to the control of anti-aging. In osteoarthritis (OA), hyaluronic acid in the cartilage matrix is reduced, resulting in a decrease in α-helices. It is known that increasing the ratio of α-helices and hyaluronic acid changes the secondary structure of proteins and improves osteoarthritis symptoms [4]. Cartilage tissue with a tonic α-helix is considered to have superior mechanical properties compared to flexible random coils.

The TMJ is one of the most common sites for osteoarthritis, and emerging evidence indicates that abnormal remodeling of mandibular condylar subchondral bone occurs during the early stages of TMJ-OA. TMJ-OA is associated with degeneration and deformation of articular cartilage and articular disks, and its main symptoms include narrowed joint range of motion, jaw movement pain, and joint noise [5,6,7]. Chen et al. reported that senescence-accelerated mice (SAMs) develop OA of the TMJ early and at a high frequency [8]. When comparing a TMJ-OA model (SAM) with wild-type mice of the same age, the mandibular condyle cartilage was thinned, chondrocytes were scattered in the intercellular space, and cartilage matrix proteoglycan production decreased [9,10,11]. Osteoarthritis of the joint is closely associated with aging; however, little is known about the age-related degeneration in the mandibular condylar cartilage of the TMJ on the molecular scale.

Naked mole-rats secrete high-molecular-weight hyaluronic acid, which protects cartilage from mechanical stress and prevents the onset of OA [12]. High-molecular-mass hyaluronic acid protects chondrocytes, inhibits degenerative changes in articular cartilage, and has been shown to be effective in maintaining joint homeostasis [13]. Furthermore, high-molecular-mass hyaluronic acid is reported to have a life-extending effect by strengthening intestinal barrier function and preventing the oxidation of immune cells [14].

Unlike hyaluronic acid, which is commercially available, high-molecular-mass hyaluronic acid of naked mole-rats readily forms robust gels without the need for chemical cross-linking.

Previous analyses of high-molecular-mass hyaluronic acid have been inconclusive, and it is difficult to visualize the spatial hierarchical structure of high-molecular-mass hyaluronic acid in living tissues [15,16].

High-molecular-mass hyaluronic acid has been difficult to identify, and its biokinetics in various tissues remain unclear. Essendoubi et al. reported that Raman spectroscopy could nondestructively evaluate the permeation of hyaluronic acid with various molecular weights in the skin layers [17]. Raman spectroscopy is sensitive to molecular vibration and can nondestructively identify and quantitatively analyze the constituent components in tissues, while Raman imaging enables the determination of compositional distribution at micrometric resolutions. Raman spectroscopy can simultaneously analyze multiple molecular species (polysaccharides, lipids, proteins, amino acids, etc.) and visualize their localization without staining. At the same time, infrared spectroscopy, a method that can analyze molecular information at the functional group level, was performed. Infrared spectroscopy and Raman spectroscopy are complementary, and information that cannot be obtained using either analysis alone can be obtained in more detail and accurately by performing the two analyses together. Therefore, in this study, we used synchrotron radiation based on Fourier transform infrared spectroscopy (SR-FTIR) and Raman spectroscopy with the aid of AI technology to visualize the molecular structure and analyze high-molecular-mass hyaluronic acid and the secondary structure of proteins in the TMJ of naked mole-rats and aged mice.

## 2. Results and Discussion

### 2.1. Comparative Micro-CT Analysis Reveals Age-Related Degeneration in Mouse TMJs, but Not in Naked Mole-Rats

**Figure 2:**
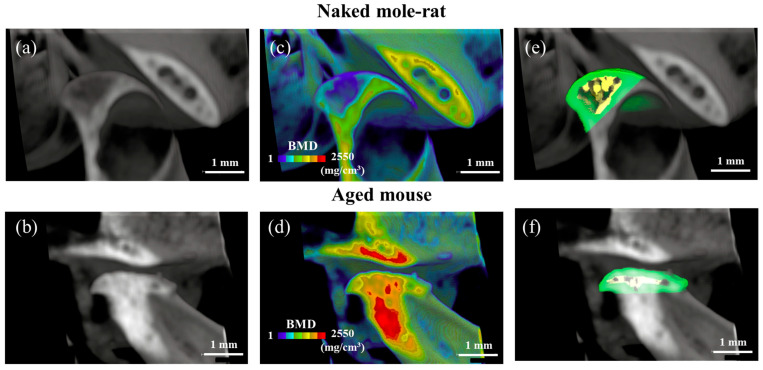
Representative three-dimensional reconstructions of (**a**,**b**) the sagittal sections of the mandibular condyle, (**c**,**d**) BMD, and (**e**,**f**) trabecular or cortical bone. Yellow: spongy bone, Green: cortical bone.

Micro-CT imaging was performed on the heads of 54-week-old naked mole-rats and mice. The roundness of the mandibular condyles of aged mice was lost, and the articular surfaces were flattened, showing a decreased cortical bone thickness of the mandibular condyle in aged mice (Figure 2b,d,f). The bone mineral density (BMD) of the mandibular condyle increased in aged mice. Conversely, the mandibular condyle of the naked mole-rats was rounded and had a spongy bone (Figure 2a,c,e). Compared to the aged mice, the cortical bone of the mandibular condyle in the naked mole-rats was thicker. These results indicate the typical findings of aging [18]. Conversely, no signs of aging were observed in the joints of naked mole-rats.

The critical change in the TMJ, associated with advancing age, is the replacement of cartilage with bone. The formation of calcified cartilage is due to a shift in cellular composition, and such alterations favor the onset of degenerative disorders of the TMJ.

### 2.2. Histological Findings

**Figure 3:**
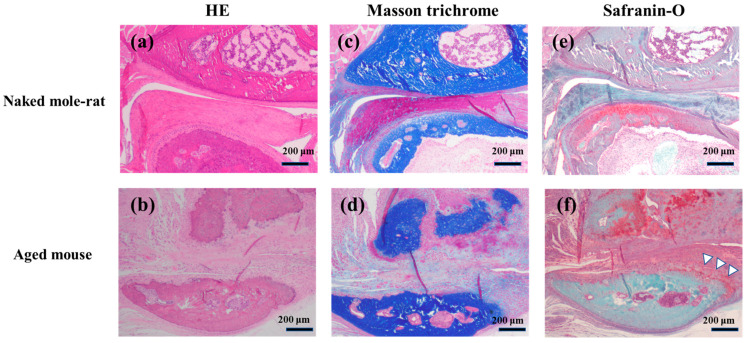
Representative micrographs of (**a**,**b**) HE, (**c**,**d**) Masson trichrome, and (**e**,**f**) safranin O-stained sagittal sections of the TMJ. Arrow heads indicate cartilaginous tissues.

Histological sections were prepared from the TMJ of 54-week-old naked mole-rats and mice. Histological findings of TMJ are shown below (Figure 3a–f). The articular disk of naked mole-rats was significantly thicker than those of aged mice. In addition, while the outline of the mandibular condyles in aged mice was markedly distorted, it was smoother in naked mole-rats, suggesting that the function of the articular disk was well maintained (Figure 3a–d). Not only the mandibular condyle of the aged mice, but also the outline of the disk and the glenoid cavity were severely distorted, the articular disk had eroded the glenoid cavity, and the joint cavity had almost completely disappeared.

In the mandibular condyle, the cartilage tissue of aged mice almost disappeared (Figure 3f). A small amount of safranin O-positive cartilage was found at the interface between the articular disk and the bone in aged mice (this is a common finding in aged mice and also in elderly people). Naked mole-rats had a considerable amount of cartilage tissue remaining near the center in the mandibular condyle (mature zone).

**Figure 4:**
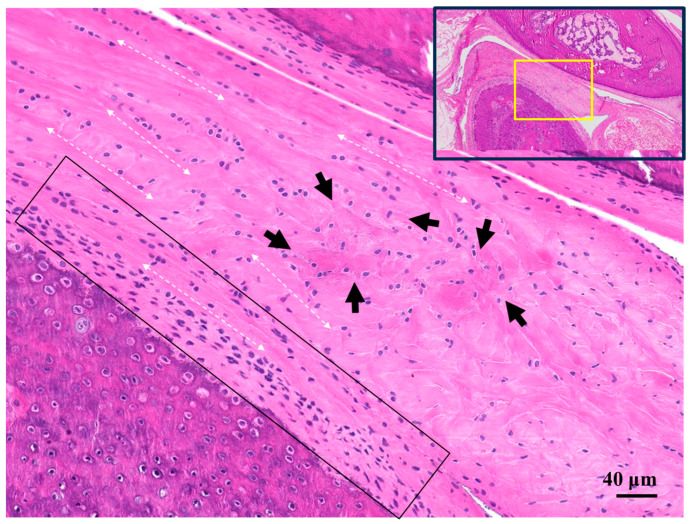
Representative microscopic images of HE-stained cross-sections of naked mole-rat articular disk (high magnification). Solid arrows and dotted arrows indicate cartilaginous tissues and collagen fiber, respectively. The black box indicates the fibrous layer of the mandibular condylar cartilage.

An enlarged image of the articular disk in the naked mole-rat showed that the collagen fibers ran in various directions in the articular disk (Figure 4). The “fibrous layer” on the surface of the mandibular condyle in naked mole-rats was thickened, and the boundary with the cartilage layer was clear (solid line). Conversely, the “fibrous layer” in aged mice was thinned, and the boundary with the cartilage layer was unclear.

The articular disk consists mainly of collagen fibers and proteoglycans constrained in the interstices of the collagen fiber mesh. In the naked mole-rat articular disk, cells were observed to be arranged horizontally (dashed line). This construction resulted in a viscoelastic response of the disk to loading and enabled the disk to play an important role as a stress absorber during function [19]. In addition, the cells in the center of the articular disk were large, spherical, bright “cartilaginous tissues” that existed in clusters (arrowhead).

In the naked mole-rat bone tissue, it is possible to distinguish between the lightly stained layer and the darkly stained deep layer just below the cartilage layer with HE staining. It is believed that bone remodeling, or metabolism, is active.

### 2.3. FTIR

**Figure 5:**
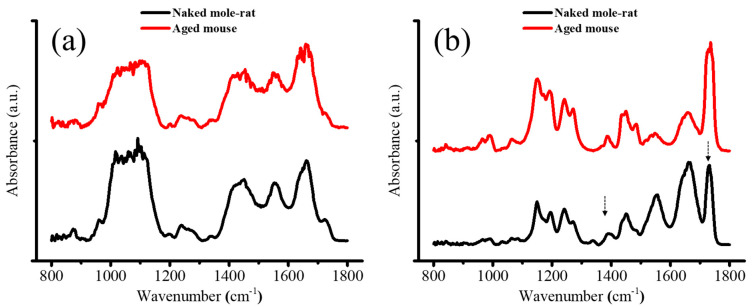
Representative FTIR spectra in the zones of the (**a**) bone and (**b**) articular disk for the naked mole-rat and aged mouse. The 1386 and 1740 cm^−1^ peaks (arrows) indicate the symmetric deformation of CH_3_ in glycosaminoglycans (GAGs) and the O-C=O stretching in phospholipids and GAGs, respectively.

**Figure 6:**
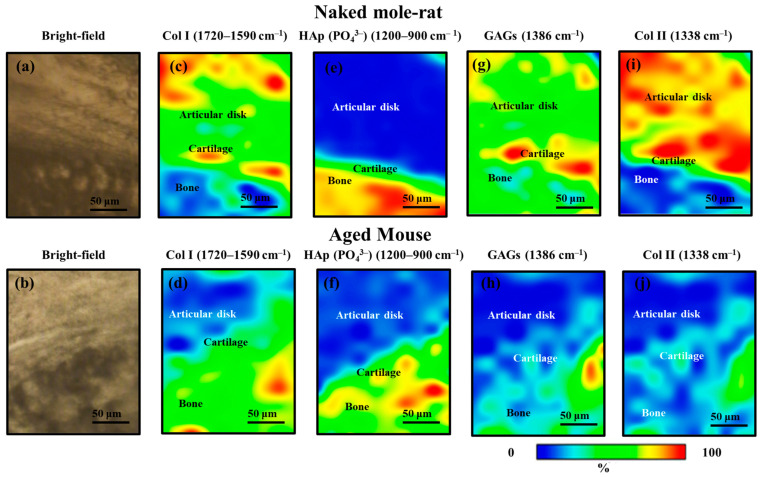
(**a**,**b**) Bright-field optical micrograph and related synchrotron radiation-based Fourier transform infrared (SR-FTIR) image of (**c**,**d**) collagen type I (Col I), (**e**,**f**) apatite (HAp), (**g**,**h**) glycosaminoglycans (GAGs), and (**i**,**j**) collagen type II (Col II) structures in a microscopic portion within the TMJ.

SR-FTIR uses synchrotron radiation, which is a high-intensity electromagnetic wave, and can obtain a signal-to-noise ratio spectrum, making it useful for analyzing the extracellular matrix (ECM) [20,21]. A comparison of the FTIR spectra taken in the zones of cortical bone of mandibular condyle and articular disk for naked mole-rats and aged mice is provided in Figure 5. As can be seen, the FTIR spectra show a strong and broad band in the range from 950 to 1100 cm^−1^ in the bone region, but not in the articular disk, as this broad band is mainly contributed by the stretching of PO_4_^3−^ in hydroxyapatite (HAp), a major constituent component of bone. No pronounced change in the profile of this band could be found between the two kinds of mice, indicating an insignificant structural alteration in condyle bone, but the reliability of this conclusion needs further validation due to the limited spectral resolution. By contrast, the articular disk exhibited two intense peaks located at ~1386 and 1740 cm^−1^, which were almost negligible in the bone region. The first peak could be attributed to the symmetric deformation of CH_3_ in glycosaminoglycans (GAGs), especially hyaluronic acid (HA) and the latter was contributed by the O–C=O stretching in phospholipids and GAGs. Moreover, compared to the aged mouse, the naked mole-rat showed the presence of a relatively small peak at 1338 cm^−1^, induced by collagen type II, as well as a stronger band located at ~1665 cm^−1^, attributed to N–C=O stretching in protein and C=C stretching in lipid. Taking advantage of these characteristic bands, SR-FTIR images could be obtained and are shown in Figure 6, which reveal pronounced variations in collagen type I (Col I) (1720–1590 cm^−1^), glycosaminoglycans (GAGs) (1386 cm^−1^), hydroxyapatite PO_4_^3−^ (HAp) (1200–900 cm^−1^), and collagen type II (Col II) (1338 cm^−1^), and their colocalization in the articular disk of the naked mole-rats (Figure 5a).

It has been debated for many years whether the articular disk was cartilage or fibrous connective tissue. In particular, the components of the articular disk differ depending on the animal. The collagen in the articular disk from the naked mole-rat was irregularly oriented, and some of it was cartilage (colocalization of GAGs and Col II).

The present results reveal that the cartilage of the mandibular condyle in naked mole-rats had a relatively large number of GAGs. It is very interesting that, unlike humans, the articular disks of naked mole-rats are composed of Col II, a component of cartilage.

### 2.4. Raman Spectroscopy

In the histological analysis, only morphological information of tissues can be obtained from stained images, but it is difficult to elucidate the molecular mechanisms of regeneration and development from the morphological information alone. The combination of Raman spectroscopy and infrared spectroscopy can comprehensively evaluate existing molecular information without damaging cells or tissues (nondestructive, nonlabeling), so their application in the field of life sciences is highly expected. By combining spectroscopic techniques that can analyze molecular-level structures with histological analysis, it was possible to simultaneously obtain morphological and positional information and molecular information, as well as analyze the spatial hierarchical structure of the ECM.

**Figure 7:**
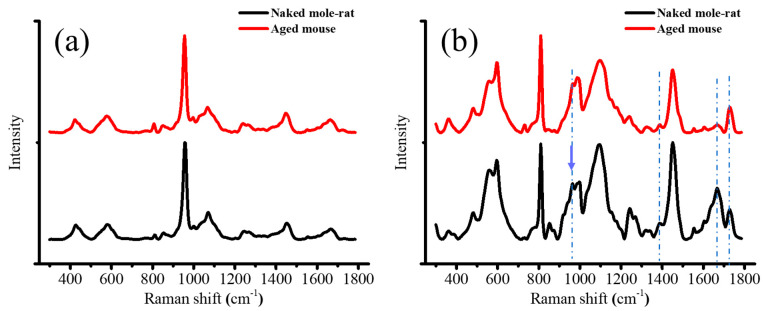
Averaged Raman spectra in the zones of the (**a**) bone and (**b**) articular disk for the naked mole-rat and aged mouse. The arrow and dotted lines indicate 960, 1389, 1670, and 1740 cm^−1^.

Figure 7a,b shows the averaged Raman spectra taken in the zones of bone and articular disk for naked mole-rats and aged mice, respectively. As can be seen, unlike the FTIR results, the symmetric stretching of PO_4_ in hydroxyapatite exhibited an overwhelming and sharp peak at 960 cm^−1^ in the bone region, while in the region of the articular disk, two sharp peaks at 810 cm^−1^ and 1740 cm^−1^ appeared, and the GAGs peak shifted to ~1389 cm^−1^ [22], and the broad band centered at ~1670 cm^−1^ was greatly altered for both kinds of mice because of the change in the constituent components. The sharp peak at 810 cm^−1^ is induced by both the backbone stretching of PO_2_ in the cell nucleus and the ring breathing of hydroxyproline, a major component of fibrillar collagen of all types [23], while the one at 1740 cm^−1^ is related to C=O stretching mainly in phospholipids. The broad band at ~1670 cm^−1^ is contributed by both C=C stretching in lipids and amide I of protein and HA.

Similarly, the band at 1670 cm^−1^ was also much weaker for the aged mice, revealing a significant reduction in protein content in the articular disk. Indeed, this observation could be confirmed by the reduction in the broad band located at ~830 cm^−1^, which was contributed by the C–C stretching of collagen.

**Figure 8:**
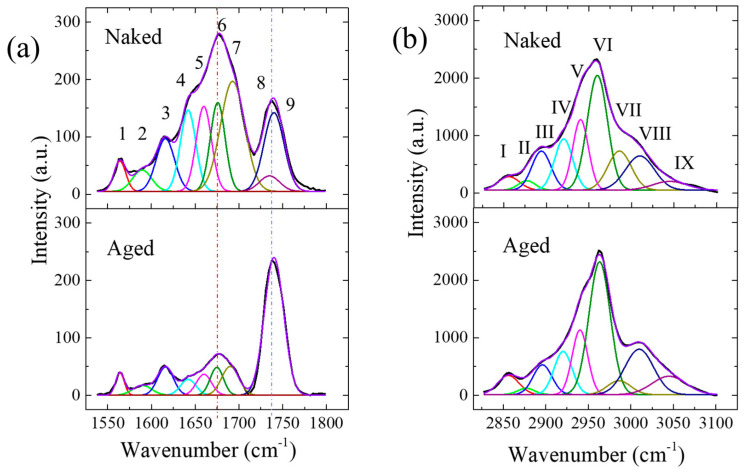
(**a**) Averaged Raman spectra in the finger-print region of the articular disk for the naked mole-rat and aged mouse. (**b**) Raman spectra in the high-frequency wavenumber interval 2700~3200 cm^−1^ after normalization of the naked mole-rat and aged mouse. The red dashed line and the blue dashed line indicate at 1660 and 1740 cm^−1^.

To retrieve more information about the secondary structure of the protein, spectral deconvolution to this broad band was performed, and the deconvoluted curves shown in Figure 8a; the assignments of relevant bands with their respective areal percentages are given in Table 1. The fitting results reveal the presence of several sub-bands in the amide I band, contributed by different types of proteins and lipids. The most intense peak located at 1675 cm^−1^ could be attributed to the β-sheet of protein in naked mole-rats, while the one at 1690 cm^−1^ was mainly caused by the disordered structure of protein and Col II. As mentioned above, the band located at ~1660 cm^−1^ could be overlapped by both C=C stretching in lipids and amide I of α-helix of col I. As can be seen from Table 1, the naked mole-rats showed a decrease in collagen type I, but an increase in the disordered structure, with respect to the aged mice. Considering the band located at 1740 cm^−1^ related to phospholipids and GAGs, the naked mole-rats exhibited an apparently broader and asymmetric peak if they were fitted by only one band. Further spectral deconvolution revealed a splitting of this band into two sub-bands and a change in their intensity ratios between these two samples because of the variations in the types of phospholipid and a higher contribution of HA in the cartilage of the naked mole-rats.

Moreover, it has been reported that Col II exhibits a very strong peak at 954 cm^−1^, which was assumed to be the most intense among those produced by various collagen types [24,25]. However, this peak was relatively weaker in the articular disk of the aged mice compared to the naked mole-rats (cf. Figure 7). It is worth noting that, as it overlaps with the *v*_1_ band of HAp, spectral deconvolution and location examination may be needed to reveal the compositional alteration at locations near the bone.

Figure 8b shows the measured and deconvoluted spectra in the range from 2825 to 3100 cm^−1^, attributed to C–H stretching in lipid and protein, and the assignments of relevant sub-bands with their respective areal percents are given in Table 2. As can be seen, the naked mole-rat showed a higher fraction of protein and a lower one of lipid, as well as a higher percentage of HA, with respect to the aged mouse.

**Figure 9:**
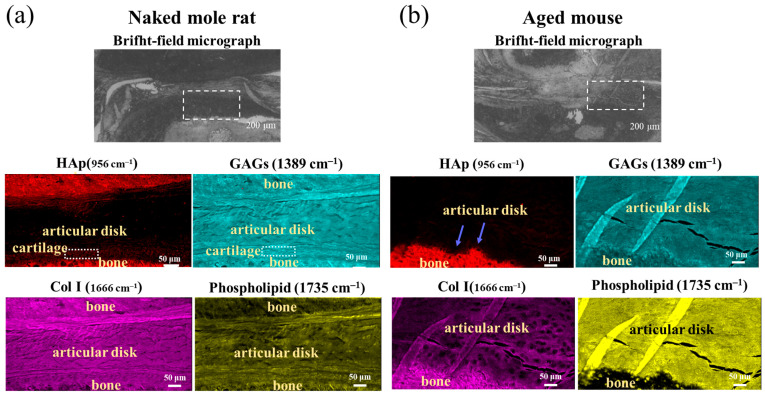
Bright-field optical micrograph and related Raman images of HAp, GAGs, Col I, and phospholipid structures in a microscopic portion within the TMJ for the (**a**) naked mole-rat and (**b**) aged mouse. The arrows and the dashed box indicate bone and cartilage, respectively.

To determine and compare the compositional variations in TMJs for naked mole-rats and aged mice, Raman analyses were performed in the same regions as those shown in Figure 3c,d on the sagittal section of TMJ of the Masson’s trichrome (MT)-stained samples; Figure 9 shows their microscope images. Taking advantage of the above characteristic peaks, respective intensity maps after background subtraction could be obtained in a selected smaller area that included the zones of bone and articular disk for each sample.

As can be seen, the variations in the 960 cm^−1^ band intensity clearly reveal the regions of bone and articular disk and their boundaries. A gradual-intensity decrease from the smooth condyle surface toward the articular disk can be observed for the naked mole-rats, but not for the aged mice, which exhibit a distorted bone surface. This might be due to the contribution of the peak at 954 cm^−1^ from the major component, Col II, in cartilage, which is consistent with the above observation of the presence of a considerable amount of cartilage near the mandibular condyle. However, for aged mice, the cartilage tissue almost disappears, so the peak intensity showed a significant reduction outside of the condyle surface. Indeed, the 1389 cm^−1^ peak intensity showed a stripe/layer-like distribution parallel to the surface of the mandibular condyle and a clear boundary of this cartilage layer, while for aged mice it exhibited a discrete distribution along the distorted bone surface and a low intensity in the articular disk. Concerning the broad bands of protein and lipid at ~1660 and 1740 cm^−1^, which consisted of many sub-bands (cf. Figure 8a), the overlap of the sub-bands significantly decreased the reliability for quantitative analyses without spectral deconvolution. Despite that, it can be seen that the protein band showed a marked intensity reduction in the articular disk of the aged mouse compared to the naked mole-rat, although the lipid band was stronger in this region.

Although Raman imaging of the TMJs could retrieve information on compositional alteration in distinct tissues, it was difficult to quantitatively capture the difference between normal tissue and pathological tissue using Raman spectroscopy from tissues composed of multiple types of molecules without a clear understanding of the assignments of the observed bands and respective contributions from different constituent components. The reasons for this also include the enormous amount of data collected using spectroscopic analysis and the need to extract microscopic spectral changes from numerous spectral deconvolution.

### 2.5. Chemometric Analysis Using Unsupervised Learning

In 2024, the Nobel Prizes in Chemistry and in Physics were related to AI; the introduction of AI into the field of life sciences is also expected [26].

It has been reported that the discrimination of heterogeneous sites using AI spectroscopic analysis is effective for applications in the field of pathology, including the identification of cancer tissue [27,28,29,30]. Using AI, it may be possible to elucidate unknown molecular groups that control hierarchical structures by extracting microscopic spectral changes that humans cannot distinguish. In histology spectroscopic analysis, AI is expected to be used as an analytical method to classify complex multivariate spectral image data systematically.

**Figure 10:**
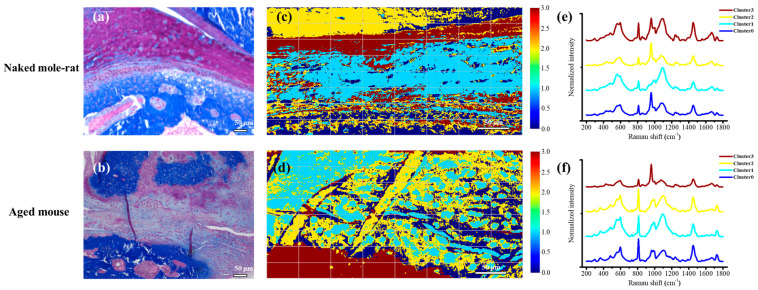
(**a**,**b**) Representative micrographs of Masson trichrome (MT) staining in sagittal sections of the TMJ. (**c**,**d**) Spatial images obtained using cluster analysis using AI, consistent with the tissue images. (**e**,**f**) Averaged spectra of the clusters obtained in (**c**,**d**) for the (**a**,**c**,**e**) naked mole-rat and (**b**,**d**,**f**) aged mouse. AI emphasizes the differences in these overall spectra, even though the differences seem small in a qualitative manner. Detailed differences are attributed to signals at ~1000 cm^−1^ and the slope from 2000 cm^−1^ to 4000 cm^−1^.

The enlarged photographs of the articular disk stained with MT are shown in Figure 10a,b. As seen in Figure 4, the naked mole-rat’s articular disk is rich in ECM. However, when the articular disk cells from the aged mouse were enlarged (Figure 10b), fairly spherical chondrocytes were observed, but, unlike those from the naked mole-rats, very little ECM (such as GAGs) was observed, suggesting that cellular activity was significantly reduced.

The cluster analysis by unsupervised learning based on an AI system was then used in an attempt to discriminate the spectra and visualize the molecules by extracting microscopic changes in the Raman spectra, and, accordingly, the spatial images could be obtained (Figure 10c,d), which matched the histological findings. In detail, the technique of uniform manifold approximation and projection (UMAP) was applied to measured spectra to detect differences. UMAP performs dimensionality reduction that preserves local structure while emphasizing the differences in information between spectra [31]. Specifically, we parameterized the image in 3D using UMAP, statistically divided the points into clusters, and reconstructed the image by assigning a color to each cluster. This characteristic of the method contributes to visualizing cells clearly without prior knowledge. To investigate the clusters formed in the reduced space, we computed average spectra from each cluster identified by UMAP, as shown in Figure 10e,f. Comparing spectra from cluster numbers 1 and 2, UMAP successfully captured significant differences and represented them in the lower-dimensional embedding. This demonstrates that this algorithm can detect and distinguish differences that are difficult to judge by human observation alone.

The results of the cluster analyses show that, in naked mole-rats, Cluster 2 (yellow) and Cluster 0 (blue) emphasized the 960 cm^−1^ Raman band contributed by HAp and represented bone with different collagen contributions (Figure 10e). Cluster 3 (brown) revealed the presence of the 940 cm^−1^ band derived from Col II and the bands at 1123 cm^−1^ and 1389 cm^−1^ derived from HA, which is characteristic of cartilage, and visualized the presence of cartilage-like tissue in the articular disk. Cluster 1 (sky blue) is thought to be fibrous tissue in the articular disk. In the AI clustering of aged mice, Cluster 3 (brown) emphasized the 960 cm^−1^ band, characteristic of bone. Cluster 1 (sky blue) revealed the absence of the sharp HAp 960 cm^−1^ band and emphasized the Raman band at 940 cm^−1^, which is a molecule derived from cartilage (Figure 10f). In this cluster, cartilage ECM was hardly formed, and hypertrophy, which is a characteristic of cellular aging, was also observed. Conversely, it was difficult to characterize Cluster 2 and Cluster 0 because the ECM hierarchical structure of aged mice was destroyed. Note that the clusters of bone (960 cm^−1^) and cartilage (940 cm^−1^) could not be sufficiently separated, possibly because the images were taken in a state in which the tissue size and signal overlapped in the depth direction. Due to the measurement principle, it is difficult to completely separate overlapping signals without deconvolution, so a certain ratio must be set as the threshold.

It was revealed that the articular disk of the naked mole-rats was composed of tissues with two types of spectra. This result suggests that cartilaginous tissues were present in the articular disk, a finding similar to that of HE staining (Figure 4). In the aged mice, chondrocytes in the mandibular condyle were scattered and ECM formation was reduced. Based on the above results, we succeeded in using chemometrics to automatically and efficiently extract molecular information contained in biological tissues from spectroscopic images.

In addition, the areas where HA was highly expressed in the naked mole-rats were different from the areas where the amide I band (1690–1620 cm^−1^) was highly expressed. This result suggests that the articular disk was composed of multiple cell groups. In the TMJ of aged mice, HA was scattered and did not constitute an ECM layer. The peak position of the amide I band was shifted to the higher-frequency side compared to naked mole-rats, confirming the dominance of β-sheets.

The high-frequency peak shift in amide I may be due to the effect of cellular aging resulting from accumulated amyloid-like aggregates of β-sheets [32].

Previous reports have shown a positive correlation between α-helix and HA in chondrocytes [7]. This is a sign of aging, and it can be inferred that the production of cartilage matrix was reduced. Conversely, it was thought that homeostasis was maintained in naked mole-rats by protecting the mandibular condyle from occlusal pressure (mechanical stress) through the articular disk, which expresses high levels of cartilage matrix.

Naked mole-rats are reported to have extremely low levels of omega-3 polyunsaturated fatty acids (PUFAs) [33]. Lipid peroxides made from PUFAs are highly toxic and destroy cell structures, and it is believed that the susceptibility of phospholipids to oxidation affects aging and longevity. In Table 1, the scattering intensity (1660 cm^−1^) of unsaturated fatty acids in aged mice was higher than that in naked mole-rats. However, this band overlapped with amide I, so the wavenumber resolution needs to be increased to analyze unsaturated fatty acids in detail.

In a report by Mitchell et al., it was speculated that phospholipids are not subject to cellular damage caused by lipid peroxides because naked mole-rats have low levels of PUFAs. However, in this study, naked mole-rats were fed raw vegetables and fruits, unlike experimental mice, so dietary considerations must be taken into account.

In 2019, the World Health Organization added an item called “aging-related” to the International Classification of Diseases (ICD-11), defining aging as a disease. This has led to a growing awareness that aging can be prevented and treated, and it is estimated that the market size of aging research will expand rapidly by 2030.

In most species, senescent cells cause irreversible aging, and release factors that damage tissues, contributing to various age-related diseases and cancer. Naked mole-rats are thought to protect themselves from the damaging effects of senescent cells by removing them [34]. Liu et al. found that monoiodoacetic acid-induced osteoarthritis of the TMJ reduced fatty acid synthesis metabolism and oxidative phosphorylation in chondrocytes [35]. In osteoarthritis of the TMJ, reactive oxygen species damage chondrocytes by activating inflammatory pathways, which caused apoptosis, dysfunction, and hypertrophy of chondrocytes and accelerated the degradation of the cartilage matrix.

This is consistent with the changes in chondrocyte metabolism during osteoarthritis [36]. Decreased oxidative phosphorylation leads to decreased ATP production, which affects the normal physiological functions of chondrocytes, such as the synthesis and repair of the cartilage matrix. Fatty acids are involved in the synthesis of chondrocyte ECM. Decreased fatty acid synthesis metabolism leads to decreased cartilage matrix synthesis, which affects the normal function of cartilage.

In aged mice, symptoms similar to those of osteoarthritis of the TMJ, such as decreased production of cartilage matrix and hypertrophy of chondrocytes, were observed by spectroscopic analysis.

Conversely, the naked mole-rats are rich in HA, which has antioxidant properties, as well as unsaturated fatty acids that promote the production of cartilage matrix, making them resistant to the development of TMJ-OA.

Aging-related quantitative and qualitative changes have been thoroughly studied in the articular cartilage of the knee, but little to no information is available on the age-related changes on the molecular scale in the cartilage, articular disks, and the subchondral bone of the TMJ. We evaluated the effect of high-molecular-mass HA on the secondary structure of proteins in naked mole-rats and aged murine joint tissues. By clarifying the molecular mechanism of anti-aging using Raman spectroscopy and FTIR, this research will lead to the development of new treatment and prevention methods for oral hypofunction and sarcopenia. Kanno et al. have successfully categorized a variety of prokaryotes accurately by combining random forest (RF) machine learning with Raman spectroscopy. Based on the feature importance scores against each wavenumber, carotenoids and membrane lipids can become bacteria-specific Raman markers and are effective in identifying prokaryotic species [37].

Data processing using AI can be easily used by nonspecialists and can be a cell analysis tool for identifying unknown dark matter hidden in cells.

Both the 2024 Nobel Prizes in Physics and Chemistry were awarded for AI-related research. Data-driven spectroscopic analysis using AI is expected to provide many breakthroughs in histology and pathology. Conversely, accumulating training data are important to improve the accuracy of AI analysis. Furthermore, when it comes to classifying cells, using a combination of single-cell analysis, RNA sequencing and spatial omics can help to discover unknown cell groups by quantifying and visualizing RNA expression levels in each cell population.

In our research, we observed a significant decrease in proteoglycans (safranin O staining) in the cartilage of the aged mice and obtained similar results to those reported by Chen et al. [18]. In aged mice, the TMJ was more susceptible to aging than the articular cartilage of the upper limbs (Supplementary Materials).

Conversely, in naked mole-rats, there was no age-related decrease in GAGs (including HA) in the mandibular condyle, and not only the mandibular condyle, but also the articular disks were maintained.

Spectroscopic analysis revealed cartilage characteristics such as type II Col and GAGs in the naked mole-rat’s articular disk. Whether cartilage is present in the articular disk is a matter of debate, as it differs depending on the animal species. The articular disks of rabbits, goats, cows, horses, and guinea pigs contain cartilage tissue, whereas mice, dogs, cats, pigs, monkeys, and humans (with pathological changes in which cartilage may appear) contain very little cartilage [38]. The TMJ disks of humans, dogs, monkeys, and rats exhibit a histological structure of fibrous connective tissue, with the majority of the cellular composition being fibroblasts. Chondrocyte-like cells are present in the TMJ disk of guinea pigs, and chondrocytes increase in number with age [39]. When cells are damaged, phospholipids may not be subject to cellular damage in the articular disk, showing strong Raman signals at 1740 cm^−1^. Recent studies have revealed that rat articular disks are morphologically fibroblastic, but contain “cartilaginous tissues” that express aggrecan and Col II collagen. Naked mole-rats are classified as rats, so the phenotype of their articular disks is thought to be consistent with previous reports [40].

Remodeling is constantly taking place in the hard tissues of the TMJ. However, when excessive load is applied to the TMJ, and the balance with the joint’s adaptive capacity is lost, and TMJ-OA develops [41,42].

Degenerative changes in the mandibular condyle due to excessive load begin in the cartilage [43], and excessive load on the TMJ increases friction within the joint. It is thought that prolonged excessive load within the joint cavity generates free radicals, which cause a decrease in HA or cause its molecular weight to decrease [44]. As low-molecular-weight HA has low lubricant, it is thought that direct contact between the articular disk surface and the cartilage surface occurs, causing sudden friction in the joint.

It has been suggested that, to prevent the progression of TMJ disorders, and to alleviate and improve symptoms such as pain and movement disorders, it is important to reduce intra-articular friction by treatments such as injecting high-molecular-weight HA into the TMJ cavity.

In this study, we clarified the details of the molecular structure of naked mole-rats by spectroscopic analysis. Conversely, to clarify the anti-aging in the TMJ of naked mole-rats, it is necessary to introduce tribology and focus on dynamic physical properties such as lubricity, wear resistance, and durability.

Zhao et al. succeeded in inducing the differentiation of human TMJ disk cells by co-culturing human iPSCs and sheep TMJ disk cells [45]. The naked-mole-rat-derived iPS cell line was established by Miyawaki et al. [46], and, in the future, it will be necessary to clarify the details of the signal pathway, the mechanical property, and the metabolite, etc., using the TMJ disk cells induced to differentiate from the naked-mole-rat-derived iPS cells.

Furthermore, naked mole-rats lack the decomposition activity of HA-degrading enzymes, and it is believed that high-molecular-weight HA accumulates in the body [47].

It has been speculated that, in the TMJ of naked mole-rats, the accumulation of high-molecular-weight HA and its antioxidant effect efficiently maintain the articular disk and mandibular condyle cartilage.

The root tips of naked mole-rat incisors extend into the TMJ, and occlusal pressure may be efficiently transmitted to the mandibular condylar cartilage. In the articular disk of naked mole-rats, collagen fibers and HA layers (cartilage-like tissues) run in various directions, which may have allowed for the efficient distribution of forceful occlusal pressure.

The naked mole-rats used in the experiment were fed vegetables and fruit, while the aged mice were fed pellets. However, for the TMJ, aging should be kept in mind, as it affects the number of teeth and the quality of food [48,49]. Furthermore, unlike laboratory mice, the naked mole-rats were fed raw vegetables and fruits, so oxidation by PUFAs and the ingested nutrients must also be taken into consideration.

In progressive condylar resorption (PCR), a degenerative lesion of the TMJ, the chemokine CCL5, which controls cell migration, increases and inflammatory changes are enhanced [50]. Ariyoshi et al. reported that the molecular weight of HA in the synovial fluid of patients with TMJ disorders was reduced and that this reduction in molecular weight promoted osteoclast differentiation and reduced bone formation, affecting bone metabolism [51]. In addition, they reported that high-molecular-weight HA had an anti-inflammatory effect and inhibited osteoclast differentiation [52].

From these results, it is proposed that high-molecular-weight HA from the naked mole-rats protects not only the TMJ, but also the entire body, and may contribute to the development of prevention and treatment of PCR.

## 3. Conclusions

The following differences were confirmed between the TMJ of naked mole-rats and aged mice.

1. The mandibular condyle of aged mice was worn down and calcified, and the articular disk disappeared. The morphology of the mandibular condyle and articular disk was maintained in naked mole-rats.
2. The articular disk of naked mole-rats had fibers with high expression of HA (cartilage layer) and collagen fibers running in various directions, which reduced occlusal pressure.

Therefore, it is believed that the combination of Raman spectroscopy and FTIR with AI will contribute to elucidating the pathology of not only TMJ disorders, but also various degenerative disorders, and to developing therapeutic drugs.

## 4. Materials and Methods

### 4.1. Animals

The Ethics Committees of Kumamoto University and Kyoto Prefectural University of Medicine approved all procedures, which were in accordance with the Guide for the Care and Use of Laboratory Animals (United States National Institutes of Health, Bethesda, MD, USA). Naked mole-rats were maintained at Kumamoto University. C57BL/6 mice were purchased from CLEA Japan, Inc. (Tokyo, Japan). The experimental animals used were 54-week-old females. Representative data from two independent experiments are shown (*n* = 2 per group).

### 4.2. Histochemical Analyses

Undecalcified resin-embedded TMJ and upper-limb joint specimens were deresinated with xylene for 60 min at 60 °C to create histological sections. The next step, MT staining, was performed as follows: deresinated sections were stained with a mixed solution (10% potassium dichromate and 10% trichloroacetic acid) for 20 min, then were treated with a solution of 1% hydrochloric acid–ethanol, stained with Ponceau fuchsin (Muto Pure Chemicals Co., Ltd., Tokyo, Japan) for 5 min, treated with 1% acetic acid, and, finally, stained with a solution of phosphotungstic acid–phosphomolybdic acid. After this, the sections were stained with Aniline Blue Solution (Muto Pure Chemicals Co., Ltd.) for 30 min and washed with a 1% acetic acid aqueous solution.

To perform safranin O (SO) staining, the sections were treated as follows: deresinated sections were stained with Mayer’s Hematoxylin Solution (Muto Pure Chemicals Co., Ltd.) for 3 min, treated with a solution of 1% acetic acid aqueous solution, stained with Fast Green Solution (Muto Pure Chemicals Co., Ltd.) for 40 s, treated with 1% acetic acid aqueous solution, and then stained with a safranin O solution (Muto Pure Chemicals Co., Ltd.) for 3 min. Following this, the sections were washed with anhydrous ethanol.

To perform hematoxylin and eosin (HE) staining, the deresinated sections were stained with Mayer’s Hematoxylin Solution for 15 min, treated with a solution of 1% hydrochloric acid–ethanol, stained with eosin solution, and washed with 99.5% ethanol. After staining, the various sections were analyzed using a fluorescence microscope (BZ-X710, Keyence, Osaka, Japan) in the confocal mode.

### 4.3. Micro-Computed Tomography (CT) Analysis

TMJ tissues were scanned with a μ-CT system TOSCANER-32300μFD (Toshiba Corporation, Tokyo, Japan) and inspeXio SMX-225CT (Shimadzu Corporation, Kyoto, Japan). The reconstructed data sets were examined using three-dimensional data analysis software (TRI/3-D-BON 64, Ratoc System Engineering Co., Tokyo, Japan).

### 4.4. Synchrotron Radiation-Based SR-FTIR

The TMJ tissues were evaluated by SR-FTIR. To characterize the content and distribution of collagen, proteoglycan and biological apatite in the ECM, SR-FTIR was performed with the Beamline15 (BL15) at the Ritsumeikan University Synchrotron Radiation Center (Kusatsu, Shiga-ken, Japan).

Sample preparation and SR-FTIR spectral analysis were performed according to a previously described procedure [20,21]. Briefly, resin-embedded tissue was sagittally sectioned into 5-μm slices, which were placed onto barium fluoride (BaF2) substrates (Pier Optics Co., Ltd., Tatebayashi, Gunma-ken, Japan) immediately after slicing. Spectra were recorded using a Nicolet™ Continuμm™ (Thermo Fisher Scientific, Waltham, MA, USA) equipped with a 250 × 250 μm^2^ liquid nitrogen-cooled MCT/A detector, a 32X/NA0.65 Schwarzschild objective, a motorized knife-edge aperture, and a Prior XYZ motorized stage coupled with a Nicolet 6700 spectrometer (Thermo Fisher Scientific, Waltham, MA, USA) equipped with a Michelson interferometer. Spectral and spatial resolutions were 0.4 cm^−1^ and 10 μm, respectively. The main chondrogenic components, such as amide I (collagen type I), GAGs, collagen type II, proteoglycan, and bone, were calculated and compared in both groups. Regarding amide I, GAGs, collagen type II, proteoglycan, and hydroxyapatite (HAp), the integrated areas were assessed in the spectral regions of 1720–1590 cm^−1^, 1390–1376 cm^−1^, 1338 cm^−1^, 1140–984 cm^−1^, and 1200–900 cm^−1^, respectively [53].

### 4.5. Raman Analyses

Raman spectroscopic imaging of TMJ tissues was performed using a Raman spectrometer (RAMANtouch, Nanophoton, Osaka, Japan). The same region as the stained images was selected and 400 × 200 Raman images were taken. The laser exposure time was 30 s per line (400 points). The spectra were then denoised by singular value decomposition (SVD) using analysis software (Raman Viewer 1.4., Nanophoton, Osaka, Japan). The distribution of bone and cartilage regions was visualized by contrast displaying the band area of the 810 cm^−1^ band, indicating C–C stretching of collagen in cartilage, and the 960 cm^−1^ band, indicating HAp in bone as marker bands.

### 4.6. Chemometric Analysis

We attempted chemometric analysis using unsupervised learning based on AI as a means of efficiently extracting information from spectroscopic images. Specifically, we applied UMAP and cluster analysis to regional spectroscopic images of the TMJ tissues to attempt spatial separation within the TMJ tissue. From the averaged Raman and FTIR spectra of each specimen, the second derivative was calculated, which was used as input data for the application of the multivariate method known as UMAP using the Material Informatics software WAVEBASE 1.0 (Toyota Motor Corporation, Toyota, Aichi, Japan). WAVEBASE extracted machine learning features using a dimensionality reduction method (UMAP). Using the material analysis software WAVEBASE, four classes were specified in advance, classification was performed, and feature extraction was performed. The spectrum of a 200 × 400, 80 k pixel image was input as data. The data obtained as hyperspectral data were subjected to dimensionality reduction using UMAP, and the feature space was classified using the Gaussian Mixture Model (GMM). The dimensions were reduced using UMAP and projected into the latent space, and the number of classifications was set arbitrarily.

## Figures and Tables

**Table 1:** Wavenumbers and vibrational assignments of the main Raman bands found in the spectrum of the TMJ band labels correspond to those shown in Figure 8a.

Band Label	Position (cm^−1^)	Principal Assignment	Percent (%)	
			Aged	Naked
**Band 1** Red	1564	C=C, N–H deformation; C–N stretching (Amide II)	23.4	20.7
**Band 2** Light green	1589	ν(C=C), olefinic stretching in lipid and ν(C–N) in hydroxyproline (collagen)	22.9	23.6
**Band 3** Blue	1616	ν(aromatic C=C) in protein (tyrosine, tryptophan)	53.7	55.8
**Band 4** Light blue	1642	Amide I, *v*(C=O) in protein segment	17.2	19.1
**Band 5** Purple	1660	Amide I, *v*(C=O) in α-helix; *v*(C=C) in unsaturated fatty acids	22.2	19.9
**Band 6** Green	1675	Amide I, *v*(C=O) in β sheet	25.5	20
**Band 7** Moss green	1690	Amide I, *v*(C=O) in disordered structure and collagen type II	35	41
**Band 8** Indigo	1735	*v*(O-C=O) of COOH in HA		
**Band 9** Dark purple	1740	*v*(O-C=O) of ester group, phospholipid		

**Table 2:** Wavenumbers and vibrational assignments of the main Raman bands found in the spectrum of the TMJ band labels correspond to those shown in Figure 8b.

Band Label	Position (cm^−1^)	Principal Assignment	Percent (%)	
			Aged	Naked
**Band I** Red	2856	vs(CH_2_) in lipid (liquid)	5.1	3.7
**Band II** Light green	2877	vs(CH_3_) in lipid (hexagonal) and protein	1.4	2.1
**Band III** Blue	2896	*v*(CH) in protein	7.1	9.7
**Band IV** Light blue	2920	*v*_as_(CH_2_) in lipid & *v*(CH) in HA	9.8	11.9
**Band V** Purple	2940	*v*_as_ (CH_2_) in protein	12.7	14.3
**Band VI** Green	2963	*v*_as_ (CH_3_) in lipid (out-of-plane chain end)	35.5	30.0
**Band VII** Moss green	2985	*v*_as_ (CH_3_) in ester and HA	4.1	11.8
**Band VIII** Indigo	3010	*v*(C=C–H) in lipid chains	16.4	12.7
**Band IX** Dark purple	3050	*v*(=C–H) aromatic stretching in lipids	7.9	3.8

## Data Availability

The original contributions presented in this study are included in the article/Supplementary Materials; further inquiries can be directed to the corresponding author.

## Supplementary Materials

The following supporting information can be downloaded at: https://www.mdpi.com/article/10.3390/gels11060414/s1, Figure S1: Representative three-dimensional reconstructions of (a,c) and the sagittal sections of (b,d) the upper limbs. Yellow: spongy bone. Figure S2. Representative micrographs of (a,b) HE, (c,d) Masson trichrome, and (e,f) safranin O-stained (SO) sagittal sections of the upper limbs. Arrow heads indicate the tidemark.

## Abbreviations

The following abbreviations are used in this manuscript:

AI	artificial intelligence
CCD	charge-coupled device
CT	computed tomography
ECM	extracellular matrix
GMM	Gaussian mixture model
HA	hyaluronic acid
HE	hematoxylin and eosin
MT	Masson’s trichrome
PCA	principal component analysis
SAM	senescence-accelerated mice
SVD	singular-value decomposition
TMD	tissue mineral density
TMJ	temporomandibular joint
UMAP	uniform manifold approximation and projection
