# Supplementary material for: Spectroscopic Analysis of the Extracellular Matrix in Naked Mole-Rat Temporomandibular Joints
Source: Gels. 2025 May 30;11(6):414. doi: 10.3390/gels11060414 (PMC12192243; doi:10.3390/gels11060414)
Supplement: Supplementary file 1 [file gels-11-00414-s001.zip › gels-3643561-supplementary.pdf]

Supplementary Material

Micro-CT and histological analyses of the upper limbs of the naked mole-rats were performed. Naked mole-rats had a higher proportion of spongy bone, giving them a "youthful" appearance. Calcification was increased in the upper limb joints of aged mice, whereas no calcification was observed in the upper limb joints of naked mole-rats. This was the same symptom as the TMJs.

Histological examination revealed that the cartilage in the joints of the upper limbs of naked mole-rats was thickened, with a clear boundary between the cartilage and bone (tide mark).

Conversely, the cartilage in the upper limb joints of aged mice was worn away, and the cartilage matrix was significantly reduced.

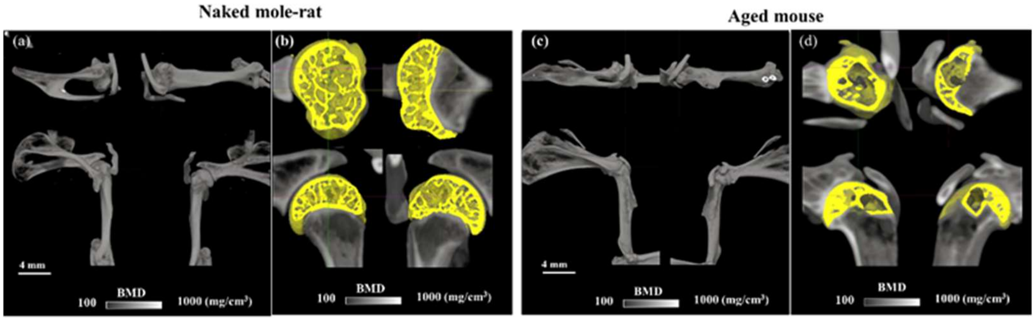

Supplementary Figure S1. Representative three-dimensional reconstructions of (a,c) and the sagittal sections of (b,d) the upper limbs. Yellow: spongy bone.

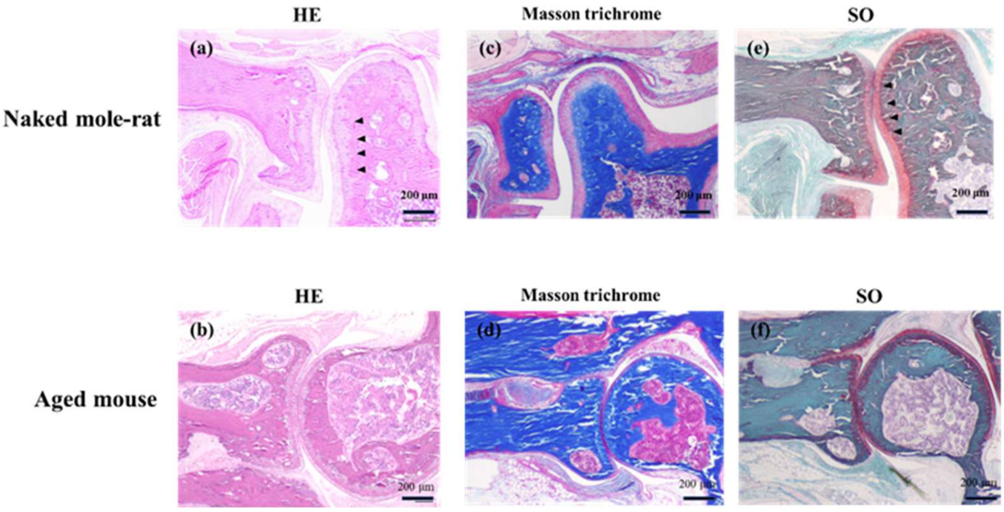

Supplementary Figure S2. Representative micrographs of (a,b) HE, (c,d) Masson trichrome, and (e,f) safranin O-stained (SO) sagittal sections of the upper limbs. Arrow heads indicate the tidemark.
